# Determinants of environmental tobacco smoke exposure in Chile: a national study using complex survey data and robust count models

**DOI:** 10.3389/fpubh.2026.1730267

**Published:** 2026-04-30

**Authors:** Francisco Novoa-Muñoz, Miguel López-Espinoza, José Leiva-Caro

**Affiliations:** 1 Departamento de Enfermería, Universidad del Bío-Bío, Chillán, Chile; 2 Centro Regional de Estudios Avanzados en Estilos de Vida Activos y Saludables, Universidad Adventista de Chile, Chillán, Chile

**Keywords:** complex survey design, environmental tobacco smoke, negative binomial regression, secondhand smoke exposure, social determinants of health, zero-inflated models

## Abstract

Environmental tobacco smoke (ETS) remains a significant public health concern in Chile despite the implementation of comprehensive smoke-free policies. This study aimed to identify sociodemographic, behavioral, and biochemical factors associated with daily ETS exposure across different environments among Chilean adults. A cross-sectional analysis was conducted using data from the 2016–2017 Chilean National Health Survey (ENS), a nationally representative survey with a complex multistage sampling design. The analytical sample included 6,233 individuals aged 15 years or older, of whom 5,520 had biochemical measurements; missing data were addressed using multiple imputation. Six outcome variables representing daily hours (0–24) of ETS exposure were analyzed across home, workplace, and other environments on weekdays and weekends. Given the high proportion of zero values (~88.5%) and evidence of overdispersion (dispersion = 6.2), Poisson, negative binomial, and zero-inflated Poisson models were formally compared using likelihood-based criteria, while quasi-Poisson models were used exclusively to assess overdispersion and the robustness of standard errors. Survey-weighted generalized linear models were fitted, and exponentiated coefficients were interpreted as mean ratios (MR). Negative Binomial models provided the best fit (AIC = 6,300 vs. 11,470 for Poisson), with consistent findings across sensitivity analyses. ETS exposure was higher among men, urban residents, and individuals reporting insomnia. Age was inversely associated with exposure (MR ≈ 0.99 per year), indicating lower expected exposure hours among older individuals. Women showed lower exposure compared to men (MR = 0.69; 95% CI: 0.55–0.87), and rural residence was associated with reduced exposure (MR = 0.61; 95% CI: 0.45–0.84). Insomnia was associated with higher exposure (MR = 1.74; 95% CI: 1.10–2.73). Descriptive analyses indicated that exposure was highest in “other environments,” particularly during weekends. These findings suggest that ETS exposure in Chile is unevenly distributed and shaped by sociodemographic and behavioral factors. Accounting for complex survey design and distributional characteristics of exposure data improves the validity and interpretability of results. Targeted public health strategies focusing on high-risk groups and environments are needed, while integrating behavioral and contextual determinants into tobacco control policies.

## 1 Introduction

Environmental tobacco smoke (ETS), also referred to as secondhand smoke, remains a major preventable risk factor for global morbidity and mortality. According to the World Health Organization, involuntary exposure to tobacco smoke is responsible for more than 1.3 million deaths annually, disproportionately affecting non-smokers, particularly women and children who share indoor environments with smokers (1). ETS exposure has been consistently associated with a wide range of adverse health outcomes, including cardiovascular disease, respiratory conditions, cancer, and, more recently, sleep disturbances and endocrine alterations (2–4).

From a conceptual perspective, ETS exposure is not merely an environmental hazard but reflects the interaction between social, behavioral, and contextual determinants. Frameworks such as the Theory of Planned Behavior highlight how attitudes, perceived norms, and behavioral intentions shape exposure patterns, particularly in shared environments (5). More broadly, ETS exposure can be understood within the exposome paradigm, which emphasizes the cumulative impact of environmental exposures across the life course and their interaction with individual susceptibility and social determinants (6, 7). This perspective is particularly relevant in understanding why exposure remains unevenly distributed across populations despite regulatory advances.

Globally, substantial progress has been made through the implementation of smoke-free policies under the WHO Framework Convention on Tobacco Control (FCTC). However, evidence indicates that ETS exposure persists, especially in private, informal, and semi-regulated environments (8, 9). Recent international studies have documented persistent exposure in workplaces and public settings, particularly in urban contexts and among vulnerable populations (10, 11). In addition, emerging research highlights the role of mental health and behavioral factors—such as sleep disturbances—in shaping exposure patterns, suggesting a more complex interplay between environmental and psychosocial determinants (3, 12).

In Latin America, ETS exposure remains a significant public health concern despite notable regulatory progress. Regional studies have shown that exposure is highly prevalent and socially patterned, with higher levels observed among younger individuals, urban populations, and socioeconomically disadvantaged groups (8, 13). More recent evidence suggests that secondhand smoke exposure continues to be widespread across the region, particularly in social and domestic settings where enforcement of smoke-free policies is limited (14, 15). Furthermore, the burden of tobacco exposure—both active and passive—remains substantial in Latin America, contributing to preventable morbidity and health inequalities (16).

Chile represents a particularly relevant context for studying ETS exposure. The country has implemented comprehensive tobacco control policies, including Law 20.660, which prohibits smoking in enclosed public spaces. Nevertheless, national data from the 2016–2017 Chilean National Health Survey (Encuesta Nacional de Salud, ENS) indicate that a considerable proportion of the population continues to be exposed to tobacco smoke in daily life. This suggests that, despite regulatory advances, exposure persists in environments that are more difficult to control, such as households and informal social spaces.

Although previous studies have identified sociodemographic determinants of ETS exposure—such as age, sex, and socioeconomic status—evidence integrating behavioral and biological factors remains limited, particularly in Latin American settings. In addition, many studies rely on analytical approaches that do not fully account for the distributional characteristics of exposure data, such as excess zeros and overdispersion, which may lead to biased or unstable estimates (17, 18). Addressing these methodological challenges is essential for obtaining more reliable and interpretable results.

This study aims to contribute to the literature by providing a comprehensive analysis of ETS exposure in Chile, integrating sociodemographic, behavioral, and biochemical factors within a nationally representative framework. In particular, we explore the role of sleep-related conditions, such as physician-diagnosed insomnia, as potential correlates of exposure, an area that has received limited attention in population-based studies. Furthermore, by applying modeling strategies that account for overdispersion and excess zeros, this study seeks to improve the robustness of epidemiological inferences regarding environmental exposure.

We hypothesize that sociodemographic factors (e.g., age, sex, urban residence), behavioral characteristics (e.g., physical activity), and health-related conditions (e.g., insomnia) are associated with variations in daily ETS exposure.

Therefore, the aim of this study was to identify factors associated with daily ETS exposure among Chilean adults, distinguishing by exposure environment (home, workplace, and other settings) and type of day (weekdays versus weekends), using data from the 2016–2017 National Health Survey.

## 2 Materials and methods

### 2.1 Study design

This cross-sectional study was conducted using publicly available microdata from the third Chilean National Health Survey (Encuesta Nacional de Salud, ENS) 2016–2017 (25). The ENS is a nationally representative epidemiological survey designed to assess health status, risk factors, and chronic disease burden in the Chilean population aged ≥15 years. Data collection was carried out between August 2016 and March 2017 through standardized household interviews, physical measurements, and laboratory testing performed by trained personnel.

The survey employed a probabilistic, stratified, multistage sampling design, including selection of geographical areas (primary sampling units), households, and individuals. The sampling strategy incorporated stratification by urban/rural area and region, with oversampling in rural and geographically extreme regions to ensure adequate representativeness. Survey weights were constructed to account for unequal selection probabilities and non-response (~20%), enabling valid population-level inference (26).

### 2.2 Participants

The analytical sample included 6,233 participants, representing the non-institutionalized Chilean population. Biochemical measurements (e.g., FT4, vitamin D3) were available for a subsample of 5,520 individuals.

To maximize statistical power and reduce bias, multiple imputation using predictive mean matching (mice package) was applied for biochemical variables with <12% missingness. Sensitivity analyses using complete-case data yielded consistent results (not shown).

All analyses incorporated sampling weights, clustering, and stratification variables provided by the ENS (expansion factor, strata, and primary sampling unit), following best practices for complex survey analysis.

### 2.3 Variables

#### 2.3.1 Outcome variables (ETS exposure)

Six count variables (range: 0–24 h/day) were analyzed to represent daily environmental tobacco smoke (ETS) exposure across settings and timeframes:

- Home exposure (weekdays): ta11_1 (HEHR)
- Home exposure (weekends): ta11_2 (HEHW)
- Work/study exposure (weekdays): ta12_1 (HEWR)
- Work/study exposure (weekends): ta12_2 (HEWW)
- Other environments (weekdays): ta13_1 (HEOW)
- Other environments (weekends): ta13_2 (HEOH)

These variables capture self-reported hours of exposure to secondhand smoke, a standard approach in population-based surveys.

#### 2.3.2 Independent variables

**Figure 1:**
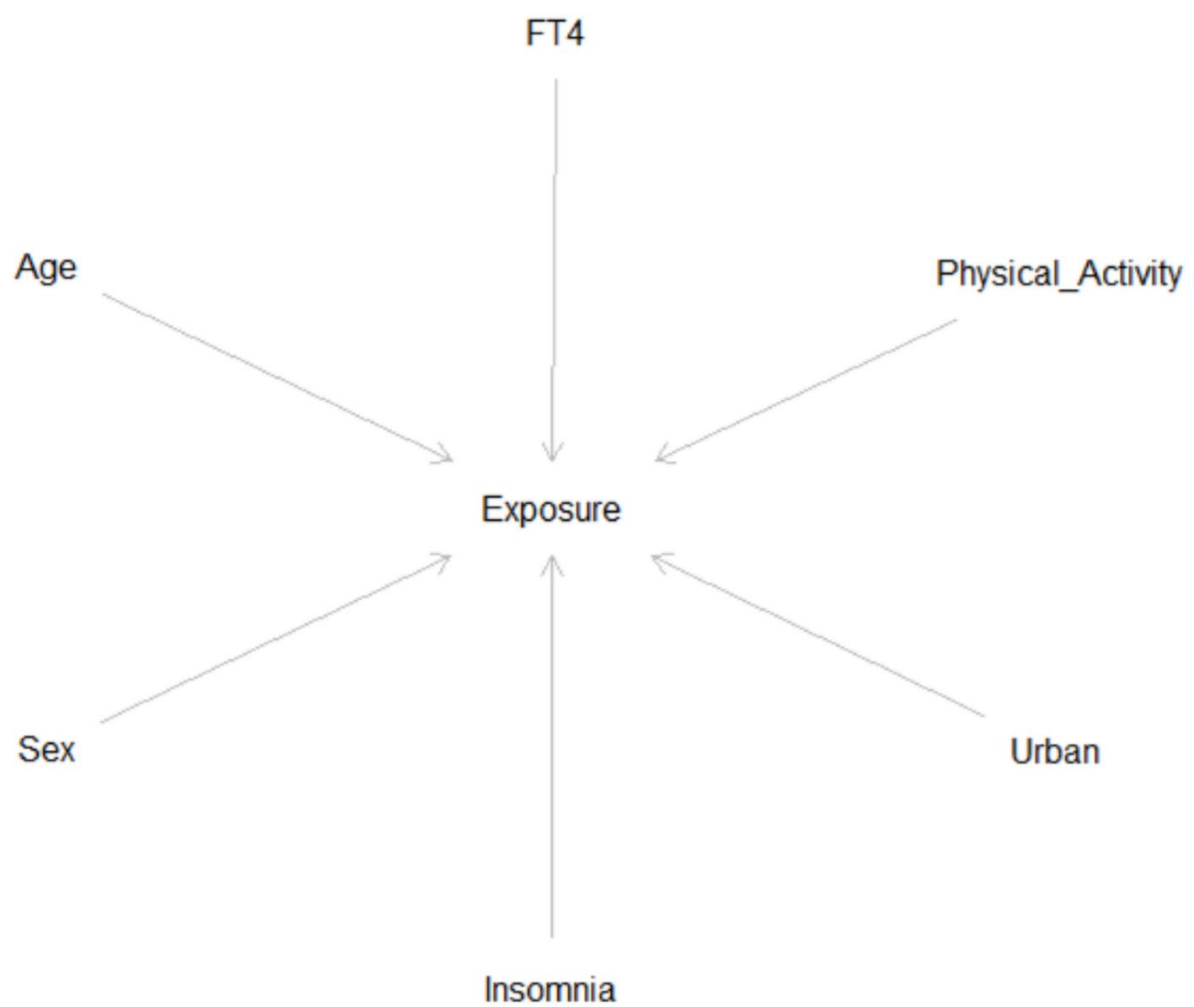
Directed acyclic graph (DAG) showing causal pathways for ETS exposure determinants.

Covariates were selected *a priori* based on epidemiological theory and prior literature on ETS exposure determinants, guided by a socioecological framework and formalized through a Directed Acyclic Graph (DAG; Figure 1).

Variables included:

- Sociodemographic (confounders)

◦ Age (continuous, years)
◦ Sex (male/female)
◦ Area of residence (urban/rural)

- Behavioral and health variables (potential mediators)

◦ Physical activity (low/moderate/high)
◦ Insomnia (yes/no), defined from medical diagnosis (ENS item *m9p22A*, recoded as binary)

- Biochemical variables (effect modifiers)

◦ Free thyroxine (FT4)
◦ Vitamin D3

Variables were retained if they met the following criteria:

1. Theoretical relevance (determinants of exposure or vulnerability)
2. Availability in ENS
3. Missingness <10–12%
4. Role in confounding control or mechanistic pathways

The DAG specified:

- Sociodemographic variables → confounders
- Behavioral variables → mediators
- Biochemical markers → effect modifiers

This approach reduces model misspecification and residual confounding.

### 2.4 Statistical analysis

#### 2.4.1 Descriptive analysis

Weighted descriptive statistics were computed to characterize the sample:

- Means and 95% confidence intervals (CI) for continuous variables
- Proportions for categorical variables

Additionally, weighted mean exposure hours by subgroup (sex, urban/rural) were estimated using svyby() to address reviewer concerns regarding baseline exposure patterns.

#### 2.4.2 Model specification

Given that outcomes represent non-negative count data with a high proportion of zeros, we implemented a multi-model strategy to ensure robust inference and appropriate model selection. Specifically, we estimated:

1. Poisson regression (baseline model)
2. Quasi-Poisson regression (to account for overdispersion in variance estimation)
3. Negative Binomial regression (primary model)
4. Zero-Inflated Poisson (ZIP) models (sensitivity analysis)

All models used a log link function, and coefficients were exponentiated to obtain Mean Ratios (MR), representing multiplicative changes in expected exposure hours.

Importantly, model selection was based exclusively on likelihood-based criteria (AIC and BIC), which are only defined for fully specified probabilistic models. Therefore, formal comparisons were restricted to Poisson, negative binomial, and zero-inflated Poisson models.

The quasi-Poisson specification was included solely to evaluate the presence and magnitude of overdispersion and to assess the robustness of standard errors, but it was not used for model comparison purposes.

#### 2.4.3 Survey-adjusted estimation

Primary analyses were conducted using:

- svyglm() from the survey package
- Incorporating:

◦ Sampling weights
◦ Stratification
◦ Clustering

Variance estimation was performed using Taylor linearization, ensuring unbiased standard errors under complex sampling.

All analyses incorporated the complex survey design of the ENS using stratification, clustering, and sampling weights (Estrato, Conglomerado, and expansion factors). To address strata with a single primary sampling unit, variance estimates were computed using the ‘adjust’ option in the survey package, a standard approach in complex survey analysis

#### 2.4.4 Model diagnostics and robustness checks

To address concerns about model instability and misspecification, we conducted:

- Overdispersion assessment

◦ Dispersion parameter >1 indicated violation of Poisson assumptions

- Zero inflation assessment

◦ Proportion of zeros ≈ 88.5%, justifying ZIP models

- Multicollinearity check

◦ Variance Inflation Factor (VIF < 2 for all predictors)

- Model comparison

◦ Akaike Information Criterion (AIC) and Bayesian Information Criterion (BIC)

- Sensitivity analyses

◦ Negative Binomial models
◦ Zero-inflated models (pscl package)

The Negative Binomial model showed substantially better fit (AIC = 6,300 vs. 11,470 for Poisson), supporting its use as the primary inferential model.

#### 2.4.5 Interpretation of estimates

Exponentiated coefficients are interpreted as Mean Ratios (MR):

- MR > 1 → higher expected exposure hours
- MR < 1 → lower expected exposure hours

For example, MR = 1.74 for insomnia indicates a 74% increase in expected exposure hours, holding other variables constant.

#### 2.4.6 Software

All analyses were conducted in R statistical software, using the following packages:

- survey (complex design analysis) (28)
- MASS (Negative Binomial models)
- pscl (zero-inflated models)
- mice (multiple imputation) (27)
- car (VIF diagnostics)

Quasi-Poisson models were used exclusively for overdispersion diagnostics and robustness checks of standard errors, and were not considered in likelihood-based model selection.

## 3 Results

Table 1 summarizes the main sociodemographic characteristics of the analyzed sample. The 2016–2017 Chilean National Health Survey (Encuesta Nacional de Salud, ENS) included a representative sample of the adult population, with the largest proportion residing in the Metropolitan Region (40.8%), followed by Biobío (11.8%) and Valparaíso (10.3%). Southern regions such as Aysén (0.6%) and Magallanes (0.9%) were less represented. Most participants lived in urban areas (88.8%), while 11.2% resided in rural settings.

**Table 1:** Weighted distribution of sociodemographic characteristics of the surveyed population.

Variable	Group	Unweighted N	Weighted N	Weighted %
Region	Arica y Parinacota	359	189,538	1.3
	Tarapacá	341	261,514	1.8
	Antofagasta	338	490,380	3.4
	Atacama	304	244,932	1.7
	Coquimbo	330	619,347	4.3
	Valparaíso	668	1,488,802	10.3
	Metropolitana	912	5,926,726	40.8
	Libertador Bdo. O’Higgins	328	738,738	5.1
	Maule	369	842,012	5.8
	Bío-Bío	661	1,710,769	11.8
	La Araucanía	322	794,180	5.5
	Los Ríos	323	327,533	2.3
	Los Lagos	346	666,982	4.6
	Aysén	327	84,825	0.6
	Magallanes y Antártica	305	132,691	0.9
Residence	Urban	5,242	12,899,655	88.8
	Rural	991	1,619,314	11.2
Age	15–24	837	2,737,931	18.9
	25–44	1815	5,414,690	37.3
	45–64	2064	4,437,480	30.6
	≥ 65	1,517	1,928,868	13.3
Sex	Male	2,315	7,131,326	49.1
	Female	3,918	7,387,643	50.9
Education	Primary	1,477	2,351,178	16.3
	Secondary	3,323	8,079,612	56.0
	Higher	1,374	3,995,592	27.7
Marital Status	Married	2,220	5,225,604	36.1
	Cohabiting without legal agreement	673	1,896,974	13.1
	Cohabiting with legal agreement	19	39,129	0.3
	Annulled	17	13,476	0.1
	Separated	426	767,720	5.3
	Divorced	208	390,826	2.7
	Widowed	674	751,092	5.2
	Single	1976	5,397,784	37.3
Income (CLP)	< 77.999	107	117,104	1.0
	78,000–134,999	473	523,044	4.3
	135,000–217,999	835	1,353,781	11.1
	218,000–295,999	723	1,619,005	13.3
	296,000–383,999	880	2,017,680	16.6
	384,000–480,999	582	1,630,232	13.4
	481,000–607,999	716	2,010,303	16.5
	608,000–764,999	232	610,057	5.0
	765,000–1,029,999	413	1,299,716	10.7
	1,030,000–1,572,999	163	462,204	3.8
	> 1,573,000	163	520,922	4.3

Chilean National Health Survey (Encuesta Nacional de Salud, ENS), 2016–2017.

The age distribution was concentrated in the 25–44 years group (37.3%), followed by 45–64 years (30.6%) and 15–24 years (18.9%), with older adults (≥65 years) representing 13.3%. The sample showed a balanced sex distribution (50.9% women, 49.1% men). Most participants had completed secondary education (56.0%), while 27.7% reported higher education and 16.3% primary education.

### 3.1 Exposure patterns across settings

Table 2 presents weighted mean exposure hours across environments and population subgroups. Exposure was highly heterogeneous, with a large proportion of individuals reporting no exposure. The highest mean exposure was observed in “other environments” during weekends, particularly among urban men.

**Table 2:** Weighted mean hours of ETS exposure (95% CI) by residence and sex.

Zone	Sex	Home_weekday	Home_weekend	Work_weekday	Work_weekend	Other_weekday	Other_weekend
Urban	Men	0.48 (0.30–0.67)	0.50 (0.31–0.68)	0.65 (0.51–0.78)	0.42 (0.31–0.53)	0.47 (0.36–0.58)	0.48 (0.38–0.59)
Rural	Men	0.24 (0.10–0.37)	0.22 (0.09–0.35)	0.36 (0.23–0.50)	0.40 (0.23–0.57)	0.23 (0.11–0.35)	0.18 (0.06–0.30)
Urban	Women	0.34 (0.25–0.44)	0.37 (0.27–0.46)	0.33 (0.25–0.40)	0.30 (0.22–0.39)	0.28 (0.21–0.35)	0.36 (0.26–0.45)
Rural	Women	0.18 (0.07–0.28)	0.15 (0.06–0.24)	0.21 (0.05–0.38)	0.09 (0.05–0.14)	0.09 (0.04–0.14)	0.16 (0.05–0.26)

Table 3 shows the distribution of key predictors across exposure groups. Individuals exposed at home tended to be older, whereas those exposed in workplaces and other environments were generally younger (*p* < 0.001).

**Table 3:** Weighted prevalence of ETS exposure by environment, sex, and residence (ENS 2016–2017).

Group	Zone	Sex	Prev
Home	Urban	Men	19.2% (16.0–22.4)
Home	Urban	Women	13.2% (11.1–15.4)
Home	Rural	Men	8.9% (5.2–12.7)
Home	Rural	Women	5.1% (3.1–7.1)
Other	Urban	Men	26.4% (22.7–30.0)
Other	Urban	Women	18.0% (15.6–20.4)
Other	Rural	Men	14.1% (8.1–20.1)
Other	Rural	Women	7.6% (3.7–11.5)
Work	Urban	Men	26.4% (22.9–30.0)
Work	Urban	Women	15.9% (13.5–18.4)
Work	Rural	Men	20.9% (14.1–27.7)
Work	Rural	Women	7.8% (4.4–11.2)

### 3.2 Model diagnostics and sensitivity analysis

The outcome variable (daily hours of ETS exposure) exhibited a high proportion of zero values (88.5%) and substantial overdispersion (dispersion parameter = 6.2). These characteristics indicate that standard Poisson models were not appropriate.

To address this, sensitivity analyses were conducted using Negative Binomial and Zero-Inflated Poisson models. Model comparison showed that the Negative Binomial model provided the best fit (AIC = 6,300), compared with the Poisson model (AIC = 11,470) and the Zero-Inflated model (AIC = 7,132).

Among likelihood-based models, the negative binomial model showed the best fit (lowest AIC/BIC), supporting its use as the primary specification.

**Figure 2:**
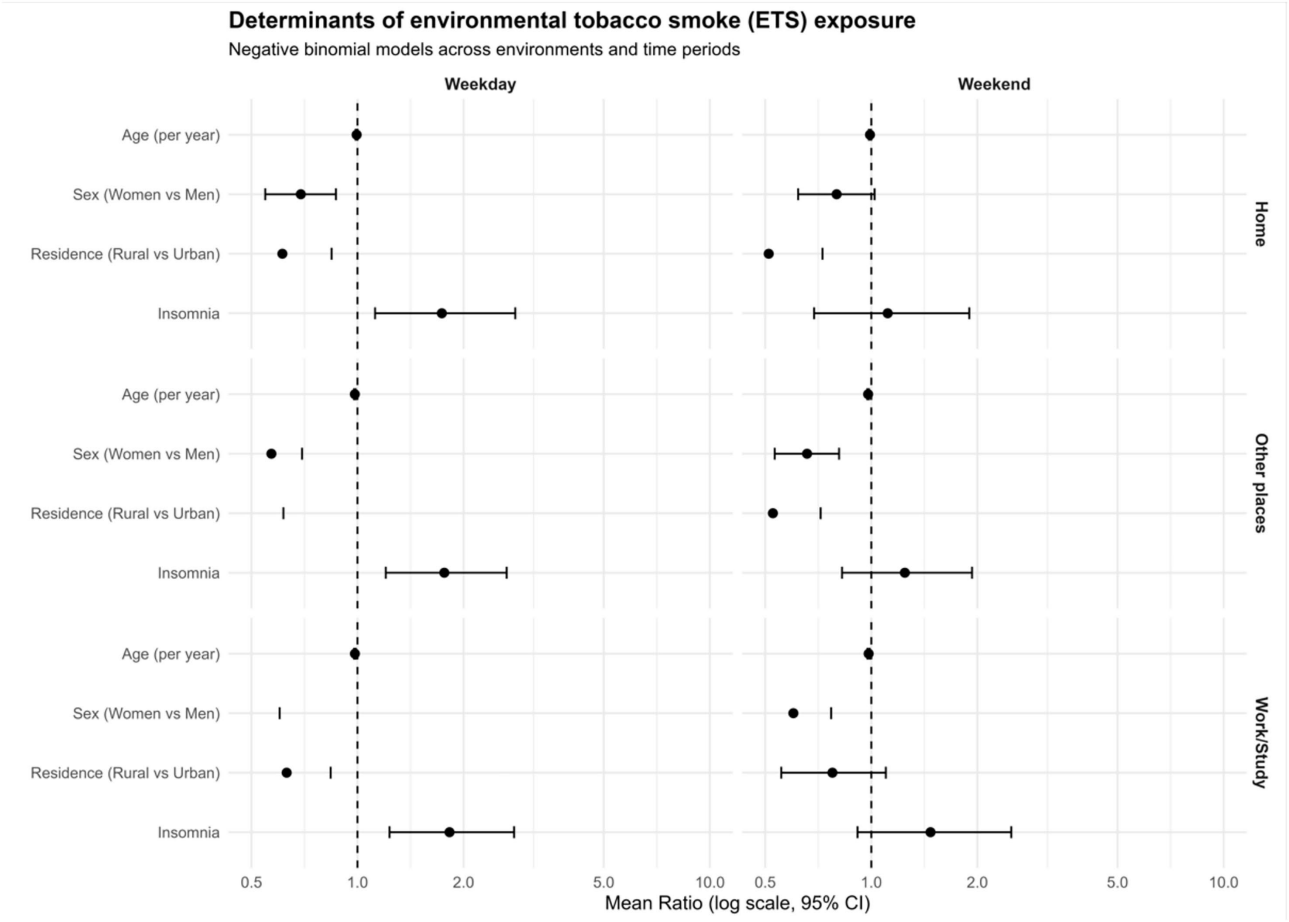
Forest plot of rate ratios from the six models.

Given these results, Negative Binomial models were used for inference, while Poisson-based models were retained only for comparability (Figure 2).

### 3.3 Factors associated with ETS exposure

Results from the Negative Binomial model are presented in Table 4.

**Table 4:** Multivariable negative binomial models of ETS exposure (mean ratios, 95% CI).

Model	Term	MR_CI
Home (Weekday)	Age (per year)	0.99 (0.99–1.00)
Home (Weekday)	Insomnia	1.74 (1.12–2.80)
Home (Weekday)	Rural vs. Urban	0.61 (0.45–0.84)
Home (Weekday)	Women vs. Men	0.69 (0.55–0.87)
Home (Weekend)	Age (per year)	0.99 (0.98–1.00)
Home (Weekend)	Insomnia	1.11 (0.69–1.90)
Home (Weekend)	Rural vs. Urban	0.51 (0.37–0.73)
Home (Weekend)	Women vs. Men	0.80 (0.62–1.02)
Other (Weekday)	Age (per year)	0.98 (0.98–0.99)
Other (Weekday)	Insomnia	1.76 (1.20–2.65)
Other (Weekday)	Rural vs. Urban	0.46 (0.34–0.62)
Other (Weekday)	Women vs. Men	0.57 (0.47–0.70)
Other (Weekend)	Age (per year)	0.98 (0.97–0.98)
Other (Weekend)	Insomnia	1.25 (0.83–1.93)
Other (Weekend)	Rural vs. Urban	0.53 (0.39–0.72)
Other (Weekend)	Women vs. Men	0.66 (0.53–0.81)
Work (Weekday)	Age (per year)	0.98 (0.98–0.99)
Work (Weekday)	Insomnia	1.82 (1.23–2.78)
Work (Weekday)	Rural vs. Urban	0.63 (0.48–0.84)
Work (Weekday)	Women vs. Men	0.49 (0.40–0.60)
Work (Weekend)	Age (per year)	0.98 (0.98–0.99)
Work (Weekend)	Insomnia	1.47 (0.91–2.50)
Work (Weekend)	Rural vs. Urban	0.78 (0.56–1.10)
Work (Weekend)	Women vs. Men	0.60 (0.47–0.77)

Older age was consistently associated with lower exposure (IRR = 0.994; 95% CI: 0.989–1.000), indicating a small but systematic decrease in exposure with increasing age.

Women showed significantly lower exposure compared to men (IRR = 0.691; 95% CI: 0.548–0.869). Similarly, individuals living in rural areas had lower exposure than those in urban settings (IRR = 0.612; 95% CI: 0.446–0.840).

In contrast, individuals with physician-diagnosed insomnia exhibited significantly higher exposure levels (IRR = 1.74; 95% CI: 1.10–2.73).

### 3.4 Interpretation of predicted exposure

Model-based comparisons suggest meaningful differences between population profiles. For example, younger urban men with insomnia tend to experience substantially higher exposure compared to older rural women without insomnia. However, given the skewed distribution of the outcome and the high proportion of zero values, these estimates should be interpreted as indicative patterns rather than precise predictions.

### 3.5 Consistency and robustness of findings

Across models and exposure settings, age, sex, urban residence, and insomnia emerged as consistent correlates of ETS exposure. Importantly, effect estimates obtained from Negative Binomial models were substantially more stable than those from initial Poisson-based analyses, which produced inflated estimates due to sparse data and excess zeros.

These findings support the interpretation that ETS exposure is unevenly distributed across population subgroups, with higher exposure observed among younger individuals, men, urban residents, and those reporting insomnia.

## 4 Discussion

This study provides nationally representative evidence on environmental tobacco smoke (ETS) exposure across multiple settings in Chile, using data from the 2016–2017 National Health Survey. By explicitly accounting for the distributional characteristics of exposure—namely a high proportion of zero values and substantial overdispersion—our analyses offer a more robust assessment of factors associated with daily ETS exposure, consistent with recommendations for modeling overdispersed health data (18, 19).

A key finding is that ETS exposure is not uniformly distributed across the population but is instead concentrated in specific subgroups. In particular, younger individuals, men, and those living in urban areas consistently exhibited higher exposure levels. These patterns align with previous research showing that urban environments concentrate sources of secondhand smoke, including shared indoor spaces and semi-regulated environments (9, 20).

Recent evidence from Latin America reinforces these findings. A pooled analysis across multiple countries in the region showed that secondhand smoke exposure remains highly prevalent and unevenly distributed, particularly among younger populations and in socially dense environments (29, 30, 33, 34). Similarly, regional public health frameworks highlight that secondhand smoke continues to be a major preventable risk factor despite existing regulations, particularly in informal and private settings where enforcement is limited.

The observed higher exposure among men is also consistent with behavioral and epidemiological evidence indicating that men have higher smoking prevalence and greater exposure through social interactions (1, 8). This pattern is particularly relevant in Latin America, where tobacco use remains disproportionately higher among men in several countries, including Chile.

Age showed a consistent inverse association with exposure, indicating that younger individuals tend to accumulate more hours of ETS exposure. This finding is consistent with regional evidence showing high levels of exposure among adolescents and young adults in Latin America, where social environments and peer behaviors play a significant role in shaping exposure patterns (31). These age-related differences likely reflect variations in daily routines, social interactions, and regulatory compliance across environments.

One of the most notable findings is the association between physician-diagnosed insomnia and higher ETS exposure. Although causality cannot be established, several mechanisms may explain this relationship. Experimental and epidemiological studies suggest that tobacco smoke exposure may impair sleep quality through respiratory and neurological pathways (4, 12). Conversely, individuals with sleep disturbances may spend more time awake or in environments where exposure is more likely. A growing body of evidence supports the association between secondhand smoke exposure and poor sleep outcomes (3, 11).

Importantly, the use of Negative Binomial models substantially improved the stability and interpretability of effect estimates compared to initial Poisson-based approaches. The high proportion of zero exposure values (88.5%) and marked overdispersion (dispersion parameter = 6.2) explain the inflated estimates observed in preliminary models. Previous methodological studies emphasize that inappropriate modeling of count data can lead to biased estimates and misleading inferences in epidemiological research (17, 18).

From a public health perspective, these findings suggest that ETS exposure remains a relevant issue even in settings with established smoke-free policies. In Latin America, recent modeling studies have shown that tobacco exposure—both active and passive—continues to generate a substantial health and economic burden, despite advances in regulation. Rather than being uniformly distributed, exposure clusters in identifiable high-risk groups, which has important implications for targeted interventions.

Furthermore, emerging regional evidence suggests that passive tobacco exposure is strongly associated with broader health outcomes, including chronic conditions, reinforcing its role as a key environmental determinant of health. In this context, the observed association with insomnia in our study suggests potential benefits from integrating tobacco control strategies with broader health promotion initiatives, including sleep health and mental well-being (21).

However, these implications should be interpreted cautiously. The cross-sectional design precludes causal inference, and associations may reflect residual confounding or reverse causality (32). Additionally, self-reported exposure may introduce measurement error, as previously documented in environmental exposure studies (22).

### 4.1 Limitations

Several limitations should be considered. First, the cross-sectional design does not allow causal directionality to be established. Second, the outcome exhibited a high proportion of zero values and substantial variability, requiring alternative modeling strategies. Although Negative Binomial models improved robustness, uncertainty remains in subgroups with sparse data.

Third, ETS exposure was self-reported rather than biomarker-based (e.g., cotinine), which may lead to misclassification (23). Fourth, despite the use of complex survey weights, potential biases due to non-response and sampling variability cannot be fully excluded (24). Finally, some predictors, such as insomnia, had relatively low prevalence, which may contribute to wider confidence intervals.

### 4.2 Future directions

Future studies should employ longitudinal designs and incorporate objective biomarkers to improve exposure assessment. Further research is also needed to clarify the mechanisms linking ETS exposure and sleep disturbances. Evaluating targeted interventions in high-risk environments—such as urban social settings and households—may provide valuable evidence for strengthening tobacco control policies.

## 5 Conclusion

This study provides nationally representative evidence on environmental tobacco smoke (ETS) exposure in Chile, highlighting that exposure is unevenly distributed across the population and associated with demographic, behavioral, and health-related factors, including age, sex, urban residence, and sleep disturbances. By explicitly accounting for overdispersion and excess zeros in exposure data, the use of Negative Binomial models improved the stability and interpretability of estimates, strengthening the robustness of the findings.

The results consistently indicate that younger individuals, men, and urban residents experience higher levels of ETS exposure across different settings. In addition, the observed association between physician-diagnosed insomnia and greater exposure suggests that ETS may be linked to broader health and behavioral contexts. While the cross-sectional design precludes causal inference, this finding highlights a potentially relevant intersection between environmental exposure and sleep-related health conditions that warrants further investigation.

From a public health perspective, these findings suggest that ETS exposure persists despite existing smoke-free policies and is concentrated in identifiable population subgroups. This pattern supports the need for more targeted and context-specific interventions, particularly in urban environments and social settings where exposure is more likely to occur. Integrating tobacco control strategies with broader health promotion efforts, including sleep and mental health, may offer additional opportunities to reduce exposure.

Overall, this study contributes updated evidence from Chile to the growing body of literature in Latin America, emphasizing the importance of considering both social and environmental determinants in understanding ETS exposure. Future research using longitudinal designs and objective exposure measures will be essential to clarify underlying mechanisms and inform more effective public health strategies.

These findings may inform the refinement of smoke-free policies and surveillance systems in Chile and similar Latin American contexts.

## Data Availability

The database is available at the following direct link: https://epi.minsal.cl/bases-de-datos/. This research used information from the Health Surveys for epidemiological surveillance conducted by the Undersecretariat of Public Health. The author thanks the Ministry of Health of Chile for providing access to the database. All results obtained from this study or research are the sole responsibility of the author and do not imply any liability on the part of the Ministry of Health.
